# Luminal-like HER2-negative stage IA breast cancer: a multicenter retrospective study on long-term outcome with propensity score analysis

**DOI:** 10.18632/oncotarget.22643

**Published:** 2017-11-24

**Authors:** Carmine De Angelis, Massimo Di Maio, Anna Crispo, Mario Giuliano, Francesco Schettini, Marta Bonotto, Lorenzo Gerratana, Donatella Iacono, Marika Cinausero, Ferdinando Riccardi, Giuseppe Ciancia, Michelino De Laurentiis, Fabio Puglisi, Sabino De Placido, Grazia Arpino

**Affiliations:** ^1^ Lester and Sue Smith Breast Center, Baylor College of Medicine, Houston, Texas 77030, USA; ^2^ Oncology Department, University of Turin, 10043 Orbassano, Italy; ^3^ Epidemiology Department, ‘Fondazione G. Pascale’ Istituto Nazionale Tumori, 80131 Naples, Italy; ^4^ Clinical Medicine and Surgery Department, University of Naples Federico II, 80131 Naples, Italy; ^5^ Department of Medical and Biological Sciences, University of Udine, 33100 Udine, Italy; ^6^ Department of Medicine (DAME), University of Udine, 33100 Udine, Italy; ^7^ Medical Oncology Unit, Azienda Ospedaliera di Rilievo Nazionale A. Cardarelli, 80131 Naples, Italy; ^8^ Advanced Biomedical Sciences Department, University of Naples Federico II, 80131 Naples, Italy; ^9^ Breast Unit, ‘Fondazione G. Pascale’ Istituto Nazionale Tumori, 80131 Naples, Italy; ^10^ Department of Clinical Oncology, CRO Aviano National Cancer Institute, 33081 Aviano, Italy

**Keywords:** breast cancer, luminal-like, chemotherapy, hormone therapy, propensity score

## Abstract

The benefit of adding chemotherapy (CT) to adjuvant hormone therapy (HT) in stage IA luminal-like HER2-negative breast cancer (BC) is unclear. We retrospectively evaluated predictive factors and clinical outcome of 1,222 patients from 4 oncologic centers. Three hundred and eighty patients received CT and HT (CT-cohort) and 842 received HT alone (HT-cohort). Disease-free survival (DFS) and overall survival (OS) were evaluated with univariate and multivariate analyses. We also applied the propensity score methodology. Compared with the HT-cohort, patients in the CT-cohort were more likely to be younger, have larger tumors of a higher histological grade that were Ki67-positive, and lower estrogen and progesterone receptor expression. At univariate analysis, a higher histological grade and Ki67 were significantly associated to a lower DFS. At multivariable analysis, only histological grade was predictive of DFS. The CT-cohort had a worse outcome than the HT-cohort in terms of DFS and OS, but differences disappeared when matched according to propensity score. In summary, patients with stage IA luminal-like BC had an excellent prognosis, however relapse and mortality were higher in the CT-cohort than in the HT-cohort. Longer use of adjuvant HT or other therapeutic strategies may be needed to improve outcome.

## INTRODUCTION

Implementation of screening programs has increased the diagnosis of early stage breast cancers in westernized countries [1–6]. Among these cases, tumors measuring ≤2 cm without lymph node involvement (N0), classified as stage IA breast cancer [7], generally have a low rate of metastatic relapse and a favorable outcome [4, 8–13]. Adjuvant hormonal treatment (HT) is the main therapeutic option for patients with stage IA “luminal-like HER2-negative” disease (“luminal-like breast cancer”), which is defined by the expression of hormone receptors (HR) and the absence of HER2 overexpression and/or amplification [14]. Clinical and tumoral features can help to characterize risk and select which patients may need CT in addition to HT. However, parameters such as tumor size and nodal status may not completely reflect the wide molecular heterogeneity seen among breast tumors even within the HR–positive subgroup [15]. Multigene prognostic tools are now beginning to guide treatment decision making. Of these, the Recurrence Score (RS) generated by the Oncotype DX® assay (Genomic Health, Redwood City, CA) gives a validated estimate of prognosis for patients with N0, estrogen receptor (ER)–positive disease if treated with tamoxifen alone [16, 17]. However, genomic tests are not always available in clinical practice, and conventional tumoral and clinical parameters still play a critical role in disease management and are the most popular tools for risk stratification in this setting. Importantly, irrespective of the criteria chosen for risk stratification, the amount of benefit associated with the addition of CT to HT is unclear.

The aim of this study was to evaluate the clinical outcome of a large cohort of patients affected by luminal-like stage IA breast cancer who received adjuvant HT with or without CT, and the prognostic value of patient- and tumor-related markers.

## RESULTS

### Population demographics and pathological features

A total of 1,222 patients from 4 Italian Oncologic Centers with luminal-like stage IA breast cancer who underwent surgery between 1996 and 2012 were identified. Data on tumor size, histological grade, ER and PgR status and Ki67 were available for 97% (1191), 96% (1,170), 99% (1,208), 98% (1,201) and 86% (1,049) of patients, respectively. The median age at the time of breast cancer diagnosis was 57 years (range:26-88 years; interquartile range, from 48 to 65 years) and 7% (87) of cases were classified T1a, 32% (381) T1b, and 61% (723) T1c. All patients included in this study had received adjuvant systemic therapy. In detail, 69% of patients (842) were treated with HT alone, and 31% (380) with CT followed by HT. The distribution of the adjuvant systemic treatments delivered in the different Oncologic Centers where patients were treated are shown in Supplementary Table 1. Seventy-eight percent (909) of tumors had a low-moderate (G1-G2) histological grade, 65% (687) had a low proliferation rate evaluated with a Ki67 threshold of 20%, and 52% (544) had a low ki67 evaluated using a threshold of 14%. With respect to HR levels, 99% (1,208) of tumors were ER-positive, with a median ER positivity of 80% (range 0-100%), and 91% (1,111) were PgR-positive, with a median PgR positivity of 70% (range 0-100%). Among PgR-positive tumors, 81% (965) had high levels of PgR (PgR≥20%).

The distribution of the demographic and pathologic characteristics of the CT vs HT cohort of patients is reported in Table 1. Age, tumor size, tumor grade, Ki67, ER and PgR expression differed significantly between the two cohorts. Patients in the CT cohort compared to those in the HT-cohort were more likely to be younger (median age 50 vs 61 years; p<0.001, respectively), and to have a larger tumor (T1c rates 81%vs. 52%; p<0.001, respectively) that had a higher histological grade (G3 rates 47% vs 12%; p<0.001, respectively), lower ER and PgR expression (ER positivity rate 96% vs 100%; p<0.001; median levels of ER expression 70% vs 90%; p<0.001; median PgR levels 50% vs 70%; p<0.001, respectively) and a higher proliferation index for both Ki67 cut-offs (Ki67 >20% 56% vs 25%; p<0.001, and Ki67 >14% 68% vs 40%; p<0.001, respectively). Not surprisingly given the distribution of baseline characteristics, the rates of luminal A breast cancer were 23% vs 48% and the rates of luminal B breast cancer were 77% vs 52% in the CT-cohort vs the HT-cohort, respectively (p<0.001).

**Table 1:** Characteristics of tumors and patients at baseline

	HT cohort(*N* =842)	CT cohort(*N*=380)	*P*-value
**Age**			
Median (range)	61 (28-88)	50 (26-76)	**<0.001**
<50 yrs	178 (21%)	179 (47%)	**<0.001**
≥50 yrs	664 (79%)	201 (53%)	
**Tumor size (*N*** **=1,191)**			
T1a	81(10%)	6 (2%)	**<0.001**
T1b	317 (38%)	64 (18%)	
T1c	428 (52%)	295 (81%)	
**Grade (*N*=1,170)**			
G1-2	719 (88%)	190 (53%)	**<0.001**
G3	95 (12%)	166 (47%)	
**Ki67 (*N*=1,049)**			
<20%	553 (75%)	134 (44%)	**<0.001**
≥20%	188 (25%)	174 (56%)	
≤14%	444 (60%)	100 (32%)	**<0.001**
>14%	297 (40%)	208 (68%)	
**ER**			
Positive (≥1%)	842 (100%)	366 (96%)	**<0.001**
Median (range)	90% (10-100%)	70% (0-80%)	**<0.001**
**PgR**			
Positive (≥1%)	770 (92%)	341 (90%)	**0.24**
High (≥20%)	686 (83%)	279 (75%)	**0.002**
Median (range)	70% (0-100%)	50% (0-100%)	**<0.001**
**Luminal (*N*=1040)**			
Luminal A	350 (48%)	70 (23%)	**<0.001**
Luminal B	384 (52%)	236 (77%)	

Luminal A: ER+ (>1%), PgR high (≥20%), HER 2-negative and Ki67 ≤14%; Luminal B: ER+ (>1%); PgR low (<20%) and/or Ki67 >14%.

Information about chemotherapy regimen was available for 99% of patients in the CT cohort. In detail, 58% (219) of patients received anthracycline-containing only regimens, 16% (60) received regimens based on both anthracyclines and taxanes, 2% (8) received a taxane-based only regimen, and 23% (89) received neither anthracycline- nor taxane-containing chemotherapy (Supplementary Table 2).

### Survival outcomes

**Figure 1:**
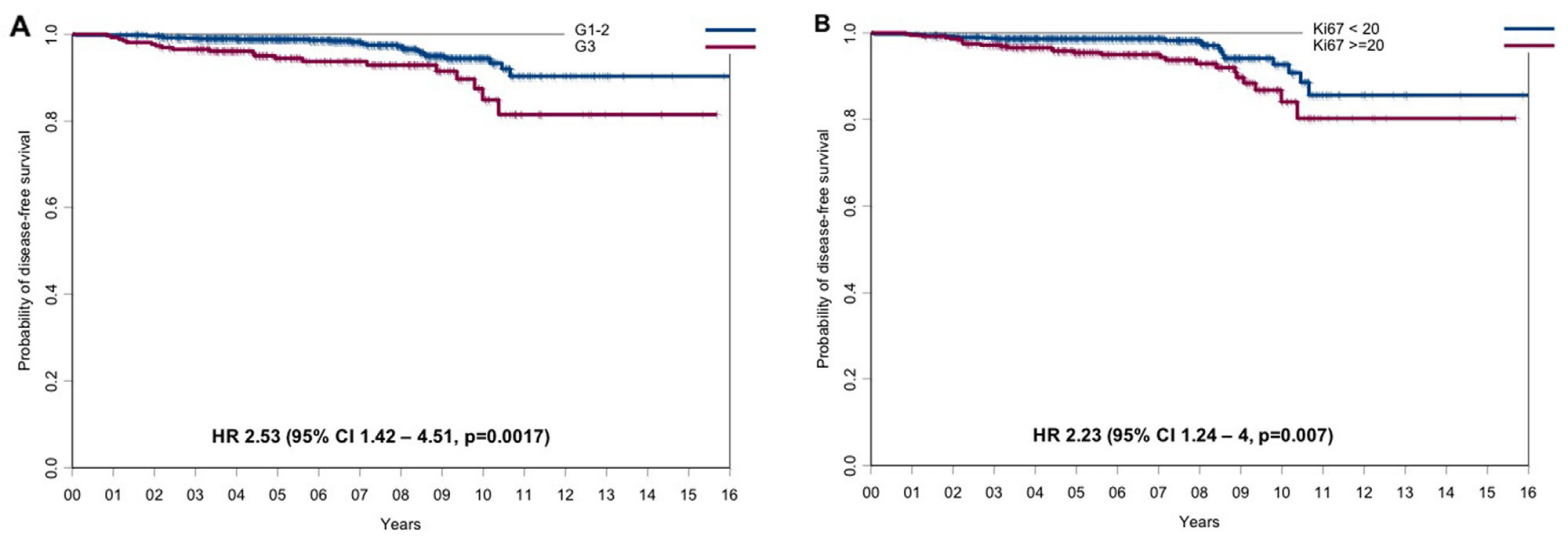
Disease-free survival according to the main prognostic factors **(A)** DFS for G1-2 vs G3 tumors and **(B)** DFS for tumors with Ki67 <20% vs tumors with Ki67 ≥20%.

Overall, with a median follow-up of 8.3 years (range: 1 to 16.4 years) in the two cohorts, the outcome of patients was very favorable with DFS rates of 97.6% and 91.5% and OS rates of 99.4% and 98.3% at 5 and 10 years, respectively. At univariate analysis, only tumor grade and proliferation were significantly associated to DFS. In detail, DFS rates at 5 and 10 years were 94.4% and 84.9% vs 98.8% and 94.4% for patients with G3 vs G1-2 breast cancer (hazard ratio=2.53, 95% CI 1.42 to 4.51, p=0.0017, Figure 1A), and 95.4% and 86.8% vs 98.6% and 92.7% for patients with Ki67 ≥20% vs Ki67 < 20% (hazard ratio=2.23, 95% CI 1.24 to 4, p=0.007, Figure 1B), respectively. Overall survival was unrelated to the other study variables. The type of relapse/event (local relapses, distant relapses and death without relapse) and their distribution in the two cohorts of patients are reported in Supplementary Table 3. Survival outcomes by histological grade, Ki67 and PgR are reported in Supplementary Table 4.

**Figure 2:**
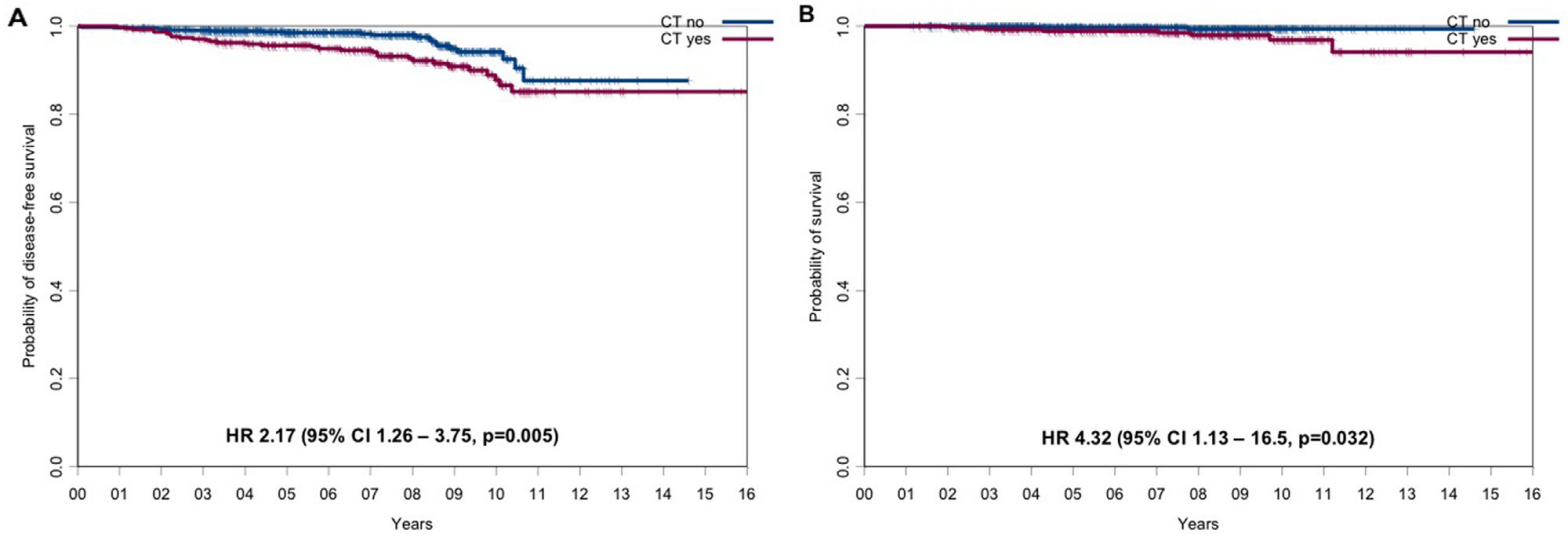
DFS and OS for the CT-cohort vs the HT-cohort **(A)** DFS and **(B)** OS for the CT-cohort vs the HT-cohort.

Not surprisingly given the distribution of baseline prognostic factors, the CT-cohort had a statistically significant worse outcome compared to the HT-cohort with regard of DFS (hazard ratio=2.17, 95% CI 1.26 to 3.75, p=0.005; Figure 2A) and OS (hazard ratio=4.32, 95% CI 1.13 to 16.5, p=0.032; Figure 2B) at 5 years and 10 years. Disease-free survival rates at 5 and 10 years were 95.6% (95% CI, 93.6%-97.8%) and 87.8% (95% CI, 83.0%-92.8%) in the CT-cohort vs 98.5% (95% CI, 97.7% to 99.4%) and 94.1% (95% CI, 91.2%-97.2%) in the HT-cohort, respectively (Table 2). Overall survival rates at 5 and 10 years were 98.9% (95% CI, 97.8%-100%) and 96.9% (95% CI, 94.3%-99.6%) in the CT-cohort and 99.7% (95% CI, 99.3%-100%) and 99.4% (95% CI, 98.5%-100%) in the HT-cohort, respectively (Table 3).

**Table 2:** DFS rates for HT-cohort vs CT-cohort

Cohort	N. of patients	N. of events	DFS rates at 5 years (95% CIs)	DFS rates at 10 years (95% CI)	Hazard Ratio(95% CI)CT vs HT
HT-cohort	842	23	98.5%	94.1%	2.17 (1.26 - 3.75) p=0.005^*^
			(97.7%-99.4%)	(91.2%-97.2%)	
CT-cohort	380	32	95.6%	87.8%	
			(93.6%-97.8%)	(83%-92.8%)	
Whole population	1,222	55	97.6%	91.5%	
			(96.6%-98.6%)	(88.6%-94.4%)	

^*^Univariate log rank test.

**Table 3:** OS rates for HT vs CT-cohort

Cohort	N. of patients	N. of events	OS rates at 5 years (95% CI)	OS rates at 10 years (95% CI)	Hazard Ratio(95% CI)CT vs HT
HT-cohort	842	3	99.7%	99.4%	4.32(1.13-16.5) p=0.032^*^
			(99.3%-100%)	(98.5%-100%)	
CT-cohort	380	9	98.9%	96.9%	
			(97.8%-100%)	(94.3%-99.6%)	
Whole population	1,222	12	99.4%	98.3%	
			(99.0%-99.8%)	(96.9%-99.7%)	

^*^univariate log rank test.

**Figure 3:**
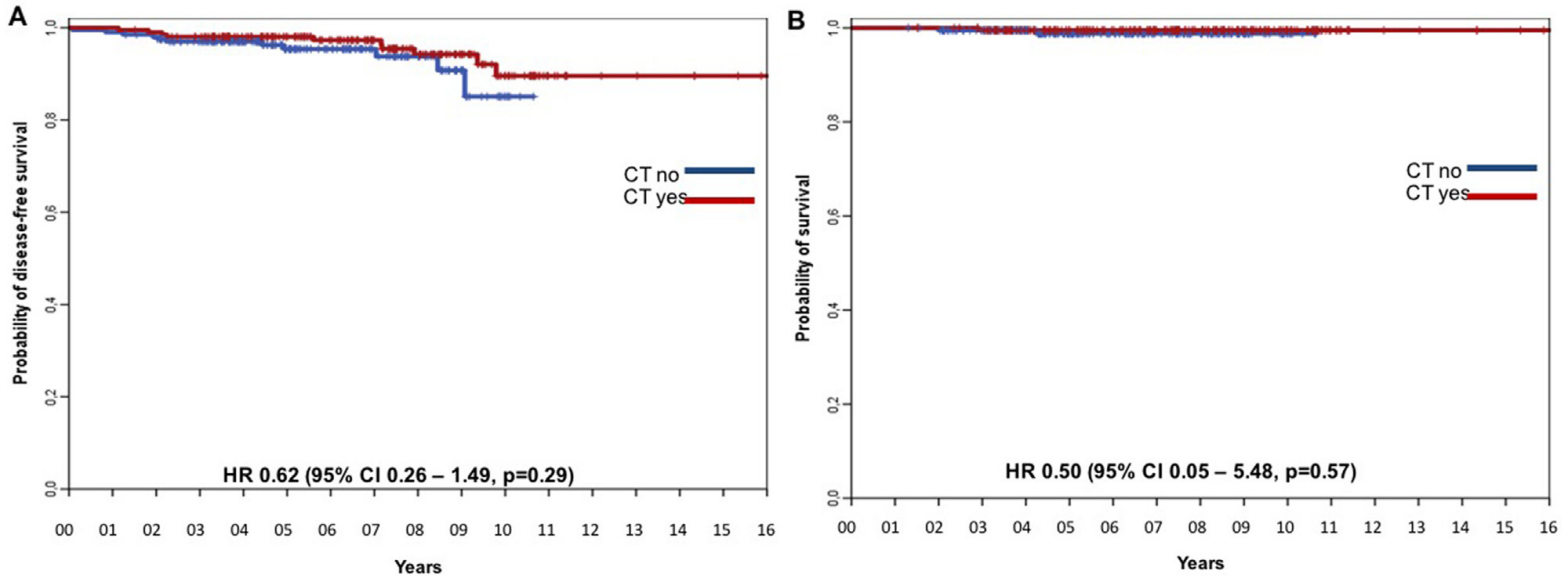
DFS for the CT-cohort vs the HT-cohort matched by propensity score **(A)** DFS and **(B)** OS for the CT-cohort vs HT-cohort matched by propensity score with Ki67 as continuous variable.

To evaluate in greater detail the impact of adjuvant treatment choice on clinical outcomes in a more homogeneous population, we analyzed survival outcome in a subgroup of 408 patients matched by propensity score (204 patients from the CT-cohort and 204 patients from the HT-cohort). The baseline characteristics of patients with luminal-like stage IA breast cancer matched by propensity score are reported in Supplementary Table 5. The items matched were patient's age, tumor size category, histological grade, PgR expression, using a cut off of 20%, and Ki67 index, as a continuous variable. When variables were well balanced, neither DFS nor OS differed significantly between the two study cohorts (Figure 3A and 3B, respectively). We also explored the interaction between CT efficacy and Ki67 levels, and found it was not significant (data not shown); however, the number of events was too low to achieve sufficient statistical power.

### Multivariate analyses

In a multivariable analysis, stratified by Oncologic Center, that included patient's age (<50 years vs ≥50 years), tumor size (T1a vs T1b vs T1c), histological grade (G1-G2 vs G3), tumor proliferation (Ki67 as a continuous variable), PgR levels (<20% vs ≥20%) and chemotherapy treatment (yes vs no), only histological grade had an independent prognostic value in predicting DFS (hazard ratio for G3 vs. G1-2 = 2.41, 95% CI 1.10 to 5.28, p=0.028; Table 4).

**Table 4:** Multivariate analyses with Cox regression model for DFS, stratified by oncologic center

Covariates		Hazard Ratios	95% CI	*P* value
Chemotherapy	Yes vs No	0.66	0.28–1.57	0.35
Age	≥50 vs. <50	0.90	0.41–1.96	0.79
T category	T1c vs T1b vs T1a	2.00	0.97–4.14	0.06
Grading	G3 vs G1-2	2.50	1.14–5.48	**0.022**
Ki67	continuous variable	1.01	0.98–1.04	0.58
ER	continuous variable	1.00	0.98–1.02	0.87
PgR	continuous variable	1.00	0.99–1.01	0.95

## DISCUSSION

Overall, our patients with luminal-like stage IA breast cancer had an excellent prognosis with survival rates at 5 and 10 years of 99% and 98%, respectively. Risk stratification, in our patients, was based on clinical and tumor parameters. As expected, given that the decision of proposing adjuvant CT was left to the physician, patients receiving this therapy were more likely to have worse prognostic characteristics (larger tumors with a higher proliferation index, higher tumor grade and lower ER and PgR expression levels) than patients receiving HT only. Among the clinical and immunohistochemical (IHC) parameters analyzed, only tumor grade was significantly related to DFS in our patients. Interestingly, despite the use of adjuvant CT, patients in the CT cohort still relapsed and their death rate was higher than that of the HT cohort. Moreover, when all the clinical and tumor features were matched by the propensity score, the addition of CT did not significantly improve either DFS or OS compared to HT alone.

In the present study, patients’ age, tumor size, grade and proliferation, and median levels of ER and PgR expression played a critical role in disease management and guided physicians and patients in therapeutic decision-making. Interestingly, however, we found that among all the clinical parameters and IHC-defined molecular biomarkers studied, only tumor grade correlated with DFS. Tumor differentiation is a well-established prognostic factor for breast cancer, and the grade score may affect the recommendation for CT in ER-positive tumors [18]. Our data and those of others [17], support the role of tumor grade in identifying, among patients with stage IA luminal breast cancer, women who may be at a higher risk of relapse, and may eventually need adjuvant CT.

The low rate of distant recurrence observed in the patients of the HT-cohort is consistent with previous reports [11, 12, 19–21] and is also in line with a risk of relapse of approximately 1% at 5 years reported by Sparano et al. in a similar patient population at a low risk based on clinicopathological features and receiving HT alone [17].

Not surprisingly, our patients in the CT-cohort had worse clinical and tumor characteristics and worse relapse and survival rates at 5 and 10 years compared to patients in the HT cohort. Of course, our results may reflect the increased baseline risk of patients in the CT cohort. However, the net benefit of adding CT to HT could not be evaluated in our study due to the lack of randomization and the retrospective nature of the analysis. In any event, when the patients’ baseline and tumor characteristics were matched by the propensity score, the addition of CT to HT did not further improve either DFS or OS. A recent meta-analysis [22] demonstrated that new generation taxane-plus-anthracycline chemotherapy regimens reduced the 10-year risk of death from breast cancer by about a third. However, as relatively few patients in the trials included in the meta-analysis had small, well-differentiated luminal A-like tumors, no definitive conclusions can be drawn about the effects of CT on such low-risk tumors.

The predominant use of older taxane-free chemotherapy regimens in the present study (only 16% of patients had received taxane and anthracycline regimens) and the relative endocrine resistance of these largely luminal B tumors may also have influenced patients’ outcome. Furthermore, data from recent neoadjuvant studies show that patients with ER-positive tumors are less likely to achieve a complete response to chemotherapy [23–26] which suggests that ER positivity can also affect the proportional risk reduction seen with adjuvant chemotherapy. New generation taxane-plus-anthracycline chemotherapy regimens, longer duration of endocrine therapies, new molecules able to more effectively block the ER pathways or other critical cell checkpoints such as cyclin inhibitors may be required to further improve survival in this cohort of patients. In this context, it is feasible that genomic tests [21, 27, 28] will help to better stratify patients according to different risk of recurrence categories [29] and define the best adjuvant strategy.

Our study has several limitations. It is a multicenter retrospective evaluation, treatment allocation was not randomly assigned and pathology was not centralized. However, the homogeneity of the patient population, propensity score matching and multivariate analysis may counteract these limitations.

In conclusion, we confirm the overall excellent prognosis of stage I luminal breast cancer. We did not identify any molecular or clinical parameters other than tumor grade able to predict patient outcome. Notably, our study shows that low-risk ER-positive disease may be treated with HT alone and, because further risk reduction from adding CT, if any, will not be large in absolute terms, this therapeutic strategy should be weighed against its toxicity. Gene expression profiling may provide additional information to better select these patients in daily clinical practice. More studies are needed to improve outcome in patients with stage I luminal breast cancer who require CT because of a high-risk score at genetic testing or a worse IHC molecular profile, because chemotherapy and 5 years HT may not be sufficient.

## MATERIALS AND METHODS

### Study population

We retrospectively collected the clinical and pathological data of early breast cancer patients who underwent surgery between 1996 and 2012 in four Italian oncologic centers (University of Naples “Federico II”, National Cancer Institute “G. Pascale”, Naples, AORN “A. Cardarelli” Hospital, Naples, and the “Santa Maria della Misericordia” University Hospital, Udine).

Pathologic data on tumor size (T), nodal status (N), histological grade (G), tumor proliferation measured by Ki67 labeling, estrogen receptor (ER), progesterone receptor (PgR) and HER2/neu status of each patient were retrieved. Breast tumors were staged according to the 7^th^ edition of the American Joint Committee on Cancer Staging (AJCC) Manual [7]. The histological grade was defined using the modified Bloom–Richardson–Elston grading system [30]. The Ki67 percentage score was assessed using the MIB1 monoclonal antibody [31, 32]. Two thresholds for Ki67 positivity (14% and 20%) were considered. HR expression was considered positive in case of ER and/or PgR immunostaining greater than 1% of invasive cells. ER and PgR were analyzed as described elsewhere [33]. PgR expression was considered high at a threshold of 20%, according to the St. Gallen Consensus [14]. HER2 receptor status was evaluated by IHC and expression level 3+ staining (DAKO Herceptest) was considered positive. In case of HER2 2+ staining, fluorescence in situ hybridization, chromogenic in situ hybridization, or silver in situ hybridization was performed to identify HER2 gene amplification. All tumors were classified as luminal-like breast cancers, with luminal-like A and B distinguished according to the St. Gallen Consensus [14]. In detail, tumors were classified as luminal A-like if ER positive (>1%), PgR high (≥20%), HER2 negative and Ki67 low (≤14%) or luminal B-like if ER positive (>1%) and PgR low (<20%) and/or Ki67 high (>14%). Patients with a diagnosis of breast carcinoma in situ (Tis), or of invasive carcinoma with no invasive lesion measuring more than 1 mm (T1mic), or a follow-up shorter than 1 year were excluded from the analysis. Overall, 1,222 patients with luminal-like stage IA breast cancer were retrospectively identified: 380 patients received CT and HT (CT-cohort) and 842 HT only (HT-cohort) as adjuvant therapy. All patients received postoperative local irradiation if indicated according to current guidelines and patient comorbidities.

This study was approved by the Ethic Committees of each participant center (IRB protocol number for Coordinating Center: 178/15). Due to the retrospective nature of the study, written informed consent was waived, according to Italian Law.

### Statistical analyses

Patients were divided into two cohorts according to the adjuvant systemic treatment administered: HT alone (HT-cohort) and CT followed by HT (CT-cohort). The χ^2^ test was used to assess differences between the groups in the distribution of categorical prognostic variables [34]. Disease-free survival was defined as the time from surgery to the date of the first event, including local or distant recurrence or death, whichever occurred first. For survivors, DFS was censored at the date of the last available follow-up. Overall survival was defined as the time from surgery until the date of death (from any cause) with censoring at the date of last available follow-up. The DFS and OS distributions were estimated using the Kaplan-Meier method [35]. The log-rank test was used to assess the difference in survival distribution between the groups [36]. Multivariate Cox proportional hazard regression analysis [37] was used to assess the independent prognostic significance of the various clinical and pathological characteristics on DFS or OS. To reduce biases related to the non-random assignment of the compared treatment strategies (CT cohort vs HT cohort), we applied the propensity score methodology [38–40]. With this method, the relationship between therapy and outcome is adjusted for the likelihood that a patient has of receiving that treatment, given her baseline characteristics. In detail, the propensity score (chance of receiving chemotherapy) was estimated by a logistic regression model that included, as dependent variable, the receipt of chemotherapy and, as covariates, factors that are likely to influence the decision whether or not to administer chemotherapy (age, tumor category, grading, PgR expression category and Ki67 value). Only subjects with overlapping values of the propensity score were included in the matched groups, with a 1:1 matching between the two study cohorts, allowing a 0.2 caliper (i.e. the maximum tolerated difference in the propensity score between matched subjects). After matching, patients of the 2 groups had a similar distribution of propensity scores, and consequently the 2 matched groups are similar in terms of age, T, grading, PgR and Ki67, whereas these factors differed greatly between the two unmatched groups.

Statistical analyses were performed with S-PLUS 6.0 Professional (release 1; Insightful Corporation, Seattle, WA, USA) and IBM SPSS Statistics (release 24.0.0.0). Propensity score analysis was performed using Propensity Score Matching for SPSS, Version 3.0.4 (Thoemmes, F. 2012. Propensity score matching in SPSS. arXiv:1201.6385).

## SUPPLEMENTARY MATERIALS TABLES



## Abbreviations

BC: breast cancer
CI: confidence interval
CT: chemotherapy
DFS: disease-free survival
ER: estrogen receptor
G: tumor grading
HR: hormone receptors
HT: hormone therapy
N: lymph nodal involvement
OS: overall survival
PGR: progesterone receptor
T: tumor size
