# Supplementary material for: Luminal-like HER2-negative stage IA breast cancer: a multicenter retrospective study on long-term outcome with propensity score analysis
Source: Oncotarget. 2017 Nov 24;8(68):112816–24. doi: 10.18632/oncotarget.22643 (PMC5762553; doi:10.18632/oncotarget.22643)
Supplement: Supplementary file 1 [file oncotarget-08-112816-s001.pdf]

Luminal-like HER2-negative stage IA breast cancer: a multicenter retrospective study on long-term outcome with propensity score analysis

SUPPLEMENTARY MATERIALS

Supplementary Table 1: Distribution of adjuvant treatments according to the Oncologic Centers where patients were treated

Oncologic Center	HT n (%)	CT n (%)	Total
University of Naples “Federico II”	205 (48.7%)	216 (51.3%)	421
National Cancer Institute “G. Pascale”, Naples	161 (73.2%)	59 (26.8%)	220
AORN “A. Cardarelli” Hospital, Naples	72 (87.8%)	10 (12.2%)	82
“Santa Maria della Misericordia” University Hospital, Udine	404 (81.0%)	95 (19.0%)	499
Total	842 (68.9%)	380 (31.1%)	1,222

Supplementary Table 2: Chemotherapy drugs administered in patients with luminal-like stage IA breast cancer

Agent	Total *N*=380 (%)
Anthracyclines	219 (58%)
Taxanes	8 (2%)
Anthracyclines plus taxanes	60 (16%)
Others	89 (23%)
Missing	4 (1%)

Supplementary Table 3: Type and distribution of relapses/events in the two study cohorts

	HT-cohort (n=842)	CT-cohort (n=380)	Overall (n=1222)
**Local relapses**	9*	10*	19**
**Distant relapses**	15*	20*	35**
**Deaths without relapse**	0	3	3
**Total**	23	32	55

*1 patient had local + distant relapse
**2 patients had local + distant relapse

Supplementary Table 4: Survival outcomes of patients with luminal-like stage IA breast cancer by histological grade, Ki67 and PgR

	G1-2 (*N*=910)			G3 (*N*=261)		
	**Total No. of Events**	**5-Year % Estimate (95%CI)**	**10-Year % Estimate (95%CI)**	**Total No. of Events**	**5-Year % Estimate (95%CI)**	**10-Year % Estimate (95%CI)**
**Outcome**						
DFS	28	98.8 (99.8-99.6)	94.4 (92.0-96.9)	20	94.4 (91.5-97.5)	84.9 (76.9-93.7)
OS	6	99.6 (99.2-100)	99.1 (98.2-100)	3	98.7 (97.2-100)	98.7 (97.2-100)
	**Ki67 <20% (*N*=687)**			**Ki67 ≥20% (*N*=363)**		
	**Total No. of Events**	**5-Year % Estimate (95%CI)**	**10-Year % Estimate (95%CI)**	**Total No. of Events**	**5-Year % Estimate (95%CI)**	**10-Year % Estimate (95%CI)**
**Outcome**						
DFS	21	98.6 (97.8-99.5)	92.7 (88.4-97.2)	26	95.4 (93.2-97.7)	86.8 (80.8-93.1)
OS	5	99.3 (98.7-100)	99.3 (98.7-100)	2	99.6 (99.0-100)	97.6 (93.7-100)
	**PgR <20% (*N*=233)**			**PgR ≥20% (*N*=965)**		
	**Total No. of Events**	**5-Year % Estimate (95%CI)**	**10-Year % Estimate (95%CI)**	**Total No. of Events**	**5-Year % Estimate (95%CI)**	**10-Year % Estimate (95%CI)**
**Outcome**						
DFS	12	98.3 (96.6-100)	85.0 (74.3-97.4)	41	97.5 (96.4-98.5)	92.6 (89.8-95.4)
OS	3	99.1 (97.9-100)	99.1 (97.9-100)	9	99.5 (99.0-100)	98.1 (96.4-99.7)

Supplementary Table 5: Baseline characteristics of patients with luminal-like stage IA breast cancer matched by propensity score

	HT-cohort (*N*=204)	CT-cohort (*N*=204)	*P* value
**Age**			0.54
<50 yrs	75 (37%)	81 (40%)	
≥50 yrs	129 (63%)	123 (60%)	
**Tumor category**			0.31
T1a	7(3%)	4 (2%)	
T1b	31 (15%)	41 (20%)	
T1c	166 (81%)	159 (80%)	
**Grading**			0.30
G1-G2	138 (68%)	128 (63%)	
G3	66 (32%)	76 (37%)	
**Ki67**			0.43
<20%	113 (53%)	105 (55%)	
≥20%	91 (47%)	99 (45%)	
**PgR**			0.91
<20%	45 (22%)	46 (22%)	
≥20%	159 (78%)	158 (78%)	
